# Auto-Intoxication

**Published:** 1919-05

**Authors:** G. N. Murphy

**Affiliations:** Paducah, Ky.


### CONTRIBUTED ARTICLES.

### Auto-Intoxication and Its Treatment.

BY DR. G. N. MURPHY, PADUCAH, KY.

Some one has said that "Auto-Intoxication is a protean monster with an endless train of ills following in its wake." This definition is so good that I think its author must have been inspired when he said it.

It is man's most deadly enemy and it is as old as mankind. As its name implies, it is a self poison which results from faulty elimination of effete matter of the animal organism.

Its greatest source arises from the bowel track, although tissue waste contributes its share as well.

Prominent among the ailments which result from body toxins are epilepsy, rheumatism, gout, neuritis, chronic nephritis, diabetes, biliousness, headache, all non-contagious skin diseases, and many other body disorders too numerous to mention.

It is not responsible, of course, for those diseases that come from without the body as the contagious and infectious ones, unless it is indirectly so by lowering the resistance of the anti-bodies of the system.

Notwithstanding its universal recognition as a mischief maker, its treatment has been most unsatisfactory. The burden of this article is to give my own treatment, which is original, for auto-intoxication which I have found most efficient.

In January, 1919, I had an attack of neuritis in my left shoulder and upper left arm. From its inception it was severe and rendered my arm almost helpless. I was unable to extend it at right angle to my body or to carry it backwards and up. I could not put my collar on or off. At first I paid but little attention to treating it as I hoped for a spontaneous recovery. After waiting six or eight weeks for improvement, I found my condition no better.

I then tried the electric spark from a coil machine for more than a month, but got no relief. I consulted one of our best practitioners who recommended gelsemium in combination with some other drug, which I do not remember, but like the electric treatment proved inefficient even to modify my trouble.

I wrote to a physician of prominence in Chicago and asked him to prescribe for me. He wrote me and expressed his regret at my condition and only suggested that I have my teeth examined for pus cavities. This I did, but the dentist pronounced my teeth in a healthy condition.

I then took over my own case for treatment and set myself to thinking what the cause could be for my neuritis; this was about nine months from the beginning of the attack.

I soon concluded that it was the result of auto-intoxication. I based my conclusions on the fact that my stools had a putrid odor, my color was anaemic and had been for many years. I had attacks of heart palpitation, always accompanied with profuse sweats and a feeling of utter exhaustion. I also suffered with occasional attacks of pectrodynia in the right pectoral muscle. I had chronic eczema, all of which I believed was caused by auto-intoxication. Now the question was how shall I treat it. I had frequently tried those remedies suggested in our text-books on practice of medicine, but only with feeble, if any permanent effect. The doses recommended in the text-books are all too small and besides they are absorbed by the stomach and do not reach the bowels where the foul poison fecal matter lies, hence the only influence exerted on the toxins was in the circulation and that only to a limited degree. My idea was to find some good antiseptic that could be brought into direct mechanical contact with the poison and neutralize it before it was absorbed into the circulation, also to clear out the bowel contents and keep the alimentary track antiseptic.

It occured to me if the antiseptics were incorporated in Russian Oil it would be an ideal plan of treatment if the bowels were well evacuated at frequent intervals and the diet regulated so as to leave out largely the albuminoids which are so largely responsible for auto-intoxication. With this in mind, I formulated the following prescription:

Creosote ........................Two drams.
Free Iodine ......................Two drams.
Oil Turpentine..................One dram.
Oil of Cloves....................One dram.
Menthol .........................Fifteen grains.
American Oil (P. D. & Co.)......One pint.

Put in colored bottle as light will disintegrate the Iodine. Tablespoon night and morning followed each alternate morning with a saline flush.

I also cut out all lean meats, eggs and cheese, pastries and highly seasoned articles of food. Under this plan of treatment

I made a complete recovery not only of my neuritis but of all the other troubles of which I had been suffering named above.

A healthy pink flush returned to my cheeks and for the past eighteen months my health seems absolutely perfect. In searching for a silver mine, I discovered gold. I have since used this treatment on others with the same gratifying results.

In some cases this treatment produces a pruritis of the arms and legs and more seldom of the trunk. In such an event discontinue the treatment for a short time until the itching subsides.

Most of these antiseptics pass through the stomach and bowels and their characteristic odor can be detected in the stools, but some of them are absorbed into the general circulation and are eliminated by the kidneys, skin and lungs, hence, every tissue of the body is brought in contact with the antidote or antiseptics.

I have varied the antiseptics, leaving out some in the above formula and substituting others, for instance, using Oil of Cinnamon for Oil of Cloves and Oil of Eulyptus instead of Tinct. Iodine, sometimes using Menthol only and leaving out Thymol. Sometimes I add Carbolic Acid, one dram, but all seem to act equally well. The dose is somewhat pungent and ought to be followed with a drink of water.

I also consider liberal water drinking, two or three quarts every twenty-four hours, an important factor in promoting elimination of waste matter of the system.

I took several pints of the oil containing the antiseptics before I gave up the treatment of auto-intoxication, and even now whenever I feel any symptoms of self-poisoning, I resume the treatment until all signs of trouble disappear, which they usually do in a week. This is seldom necessary, however, as I generally go from three to five months without any signs of trouble.

I have found some patients who would not carry out the above treatment conscientiously and therefore failed to get the proper results.

All who may try the above method of treating auto-intoxication and stick to it faithfully will be amply rewarded for the trouble.

